# Lateral femoral condyle OCD lesions in children on knee MRI

**DOI:** 10.1007/s00256-026-05168-5

**Published:** 2026-02-24

**Authors:** Jie C. Nguyen, Liya Gendler, Carlos Yaya-Quezada, Julian Forero-Millan, Wondwossen T. Lerebo, J. Todd R. Lawrence, Theodore J. Ganley

**Affiliations:** 1 Department of Radiology, Section of MSK Imaging, Children’s Hospital of Philadelphia, 3401 Civic Center Blvd, Philadelphia, PA 19104 USA; 2 University of Pennsylvania, Perelman School of Medicine, 3400 Civic Center Blvd, Philadelphia, PA 19104 USA; 3 Division of Orthopedic Surgery, Children’s Hospital of Philadelphia, 3401 Civic Center Blvd, Philadelphia, PA 19104 USA

**Keywords:** Arthroscopy, Cartilage, Children, Diagnostic performance, Imaging, Knee, Lateral femoral condyle, MRI, OCD, Osteochondritis dissecans

## Abstract

**Objective:**

To systematically investigate the various MRI findings that can associate with unstable lateral femoral condyle (LFC) osteochondritis dissecans (OCD) lesions in the pediatric knee.

**Methods:**

This retrospective study included patients < 18 years who underwent knee MRI and treatment for LFC OCD lesions, between 2018 and 2025. Blinded to outcome, 2 radiologists independently and retrospectively reviewed all examinations to determine the presence of effusion, skeletal maturity, and various direct and indirect findings that can associate with lesion instability. Demographic characteristics and lesion volume were also collected. The reference standard for lesion stability included arthroscopy (*n* = 16) and in those managed conservatively, follow-up clinical and imaging evaluations (*n* = 21). Findings between stable and unstable lesions were compared.

**Results:**

Thirty-two patients (21 males, 11 females, mean age = 12.0 ± 2.0 years, range = 8.1–17.2) had a total of 37 (26 stable, 11 unstable) lesions. Older age (14.4 ± 1.9 vs. 11.9 ± 1.5 years, *P* < .01), skeletal maturity (36.4% vs. 3.8%, *P* = .02), larger lesion volume (436.4 mm^3^ vs. 230.8 mm^3^, *P* < .01), presence of overlying cartilage alteration (90.9% vs. 7.7%, *P* < .01), progeny-parent bone interface (81.2% vs. 23.1%, *P* < .01), altered surface curvature (72.7% vs. 23.1%, *P* < .01), parent bone low-signal-intensity marginal rim (81.8% vs. 11.5%, *P* < .01), and extensive perilesional marrow edema (72.7% vs. 11.5%, *P* < .01) were more common among unstable than stable lesions. Cartilage alteration had the highest discriminative performance for identifying lesion instability with sensitivity of 91% and specificity of 92% (area under the curve, AUC = .92, 95% CI = 0.81–1.00).

**Conclusion:**

Various MRI findings can associate with unstable LFC OCD lesions, but the presence of overlying cartilage alteration had the highest discriminative performance.

## Introduction

Osteochondritis dissecans (OCD) describes a pathologic condition, which is centered at the osteochondral junction that can result in an unstable subchondral fragment and disruption of the overlying cartilage, leading to separation of the progeny from the underlying parent bone, increasing the risk for premature osteoarthritis [1–3]. Despite the routine use of knee MRI examinations to diagnose these lesions and to determine lesion stability, which impacts treatment selection, no published literature currently exists that exclusively characterizes imaging findings of lesions over the lateral femoral condyle (LFC). Within the knee, LFC is the second most common anatomic location for OCD lesions, accounting for 13–32.5% of all lesions [4–7]. While a recent investigation on medial femoral condyle (MFC) OCD lesions found that indirect MRI findings are more common than direct findings for predicating lesion instability [8], the existing published literature on LFC lesions is limited to studies that also included MFC OCD lesions and without subgroup analysis.

In recent years, location-dependent differences in the development and progression of OCD lesions are increasingly recognized, which impact the spontaneous healing potential and influence the need for surgical intervention. This is because lesions at different anatomic locations are subject to different biomechanical stresses and intrinsic differences in regional vascular perfusion [3, 9–12]. In particular, LFC OCD lesions have been found to associate with genu valgus alignment and discoid lateral meniscus, both of which can lead to crowding of the lateral tibiofemoral compartment of the knee [13–17]. In contrast to MFC lesions, LFC lesions are more likely to be unstable at presentation [18], to undergo surgical treatment, and to report persistent knee pain [19]. Therefore, there is an unmet need for location-specific data that characterize the frequency of various MRI findings and determine the association of each finding with lesion stability. Thus, the purpose of the current study was to systematically investigate the various MRI findings that can associate with unstable LFC OCD lesions in the pediatric knee.

## Methods

### Patients

This study was approved by our Institutional Review Board (IRB) and performed in compliance with Health Insurance Portability and Accountability (HIPAA) regulations, with a waiver for written informed consent.

A retrospective search of the electronic medical record database was performed using Illuminate (Softek Illuminate) to identify pediatric patients (< 18 years old) who underwent knee MRI and treatment for LFC OCD lesions in the past 7 years (January 1, 2018–February 28, 2025) at our tertiary free-standing pediatric hospital. For patients with multiple MRI examinations of the same knee, only one examination was included, either the first examination for those managed conservatively or the immediate pre-treatment MRI for those who underwent arthroscopic treatment.

**Fig. 1:**
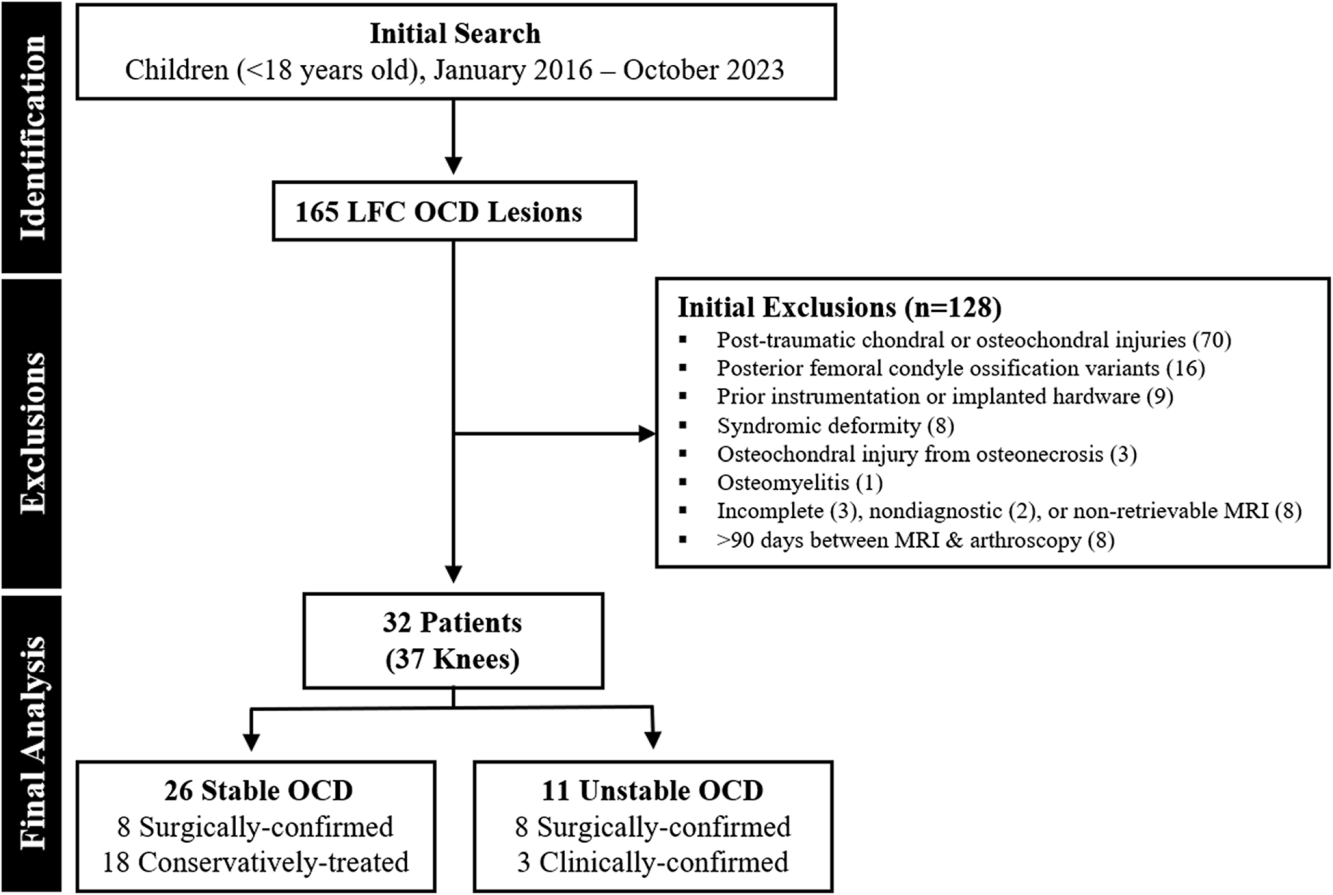
Flow diagram. Diagram shows patient identification and exclusions, which yielded the final set of lateral femoral condyle (LFC) osteochondritis dissecans (OCD) cases that were included for analysis

Knee MRI examinations performed following acute traumatic injury or for post-traumatic chondral or osteochondral injuries (*n* = 70) were excluded. Additionally, examinations on knees with isolated ossification variants (centered over the posterior femoral condyle [20]) (*n* = 16), prior instrumentation with or without retained hardware (*n* = 9), from patients with syndromic disorders (*n* = 8), osteochondral injury from osteonecrosis (*n* = 3), or osteomyelitis (*n* = 1) were also excluded. Incomplete (*n* = 3), nondiagnostic (*n* = 2), or non-retrievable MRI examinations (*n* = 8) as well as those with > 90 days between MRI and arthroscopy (*n* = 8) were also excluded (Fig. 1).

### MRI technique

MRI examinations were performed with patients in the supine position and knees in full or near-full extension using either a 1.5-Tesla (Avanto; Siemens Healthineers) or 3-Tesla magnet (Magnetom Prisma and Skyra, Siemens Healthineers) and a 15-channel phased-array knee coil (Tx/Rx coil, Siemens Healthineers, Munich, Germany). The clinical protocol included turbo spin-echo pulse sequences acquired in all 3 orthogonal planes: axial and sagittal T2-weighted fat-suppressed (TR/TE = 4500–5500 ms/45–65 ms); sagittal intermediate-weighted (non-fat-suppressed) and coronal intermediate-weighted fat-suppressed (TR/TE = 3000–4000 ms/25–40 ms); and coronal T1-weighted pulse sequences (TR/TE = 500–650 ms/10–20 ms). The field of view was 14–16 cm, the matrix size ranged between 384 × 384 and 512 × 512, and slice thickness was 3 mm with an inter-slice gap ranged between 0 and 10%.

### Review of MRI examination

Blinded to outcome, all MRI examinations were retrospectively reviewed by two board-certified, fellowship-trained radiologists—one in pediatric and musculoskeletal radiology (JCN, with 12 years of post-fellowship clinical experience) and the other in pediatric radiology with a focus in musculoskeletal imaging (LG, with 5 years of post-fellowship clinical experience). Disagreements from independent reviews were resolved through a separate consensus review.

Examinations were assessed for the presence of a knee joint effusion, regional skeletal maturity, various direct and indirect findings that can associate with lesion instability [8]. Joint effusion was characterized by the presence of hyperintense signal intensity on fluid-sensitive images that distended both medial and lateral suprapatellar gutters. Skeletal maturity was based on the patency of the distal femoral and proximal tibial growth plates. Using coronal and sagittal images, the knee was classified as *immature* if both growth plates were open (a uniform band of growth plate signal intensity that is hypointense on T1-weighted and hyperintense on fluid-sensitive pulse sequences). The knee was classified as *maturing* if there was narrowing, indistinctness, or loss of this growth plate signal that involved at least 25% of the proximal tibial growth plate, or *mature* if there was complete or near-complete closure of the proximal tibial growth plate and > 75% loss of the distal femur growth plate. Due to the relatively smaller sample size, skeletally maturing and mature knees were grouped and compared with skeletally immature knees.

**Fig. 2:**
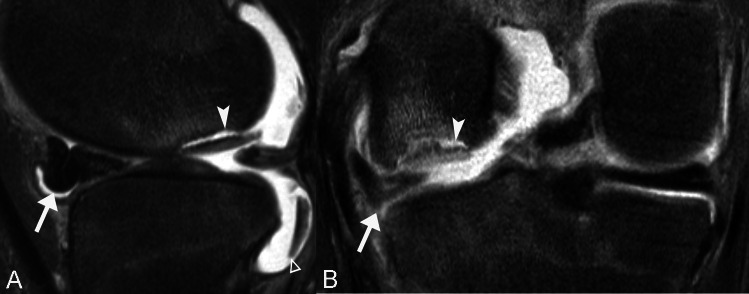
Unstable LFC OCD lesion in a 17-year-old skeletally mature female. Sagittal T2-weighted fat-suppressed (**A**) and coronal intermediate-weighted fat-suppressed (**B**) MR images show a partially detached progeny with a fluid-signal intensity progeny-parent bone interface (arrowheads). Note the intra-articular body posteriorly (triangle) and a torn, anteriorly shifted and laterally extruded discoid lateral meniscus (arrows). These findings were confirmed during arthroscopy.

Direct findings of lesion instability include an osteochondral defect, intraarticular bodies, cartilage alteration, and bone plate disruption. An osteochondral defect (or “empty crater”) resulted from a displaced progeny, leaving behind a fluid-filled concavity at the articular surface [1]. Intraarticular bodies were defined as detached chondral or osteochondral fragments. Cartilage alteration included full-thickness cartilage fissure or disruption (a band of high signal intensity) and dark cartilage lesions (or “omen sign,” a band of low signal intensity) [20–25] (Fig. 2). If cartilage alteration was detected, the quantity (single or multiple) and the location (anterior, posterior, medial, or lateral) were also recorded. Among skeletally maturing and mature patients, the subchondral bone plate is located beneath the articular cartilage and its disruption can be focal (defined as ≤ 3) or multifocal (defined as > 3 foci of disruption) [22], whereas among skeletally immature patients, the ossification front is located beneath the chondroepiphysis, which can be normally located or receded, reflecting localized endochondral ossification dysfunction [26].

**Fig. 3:**
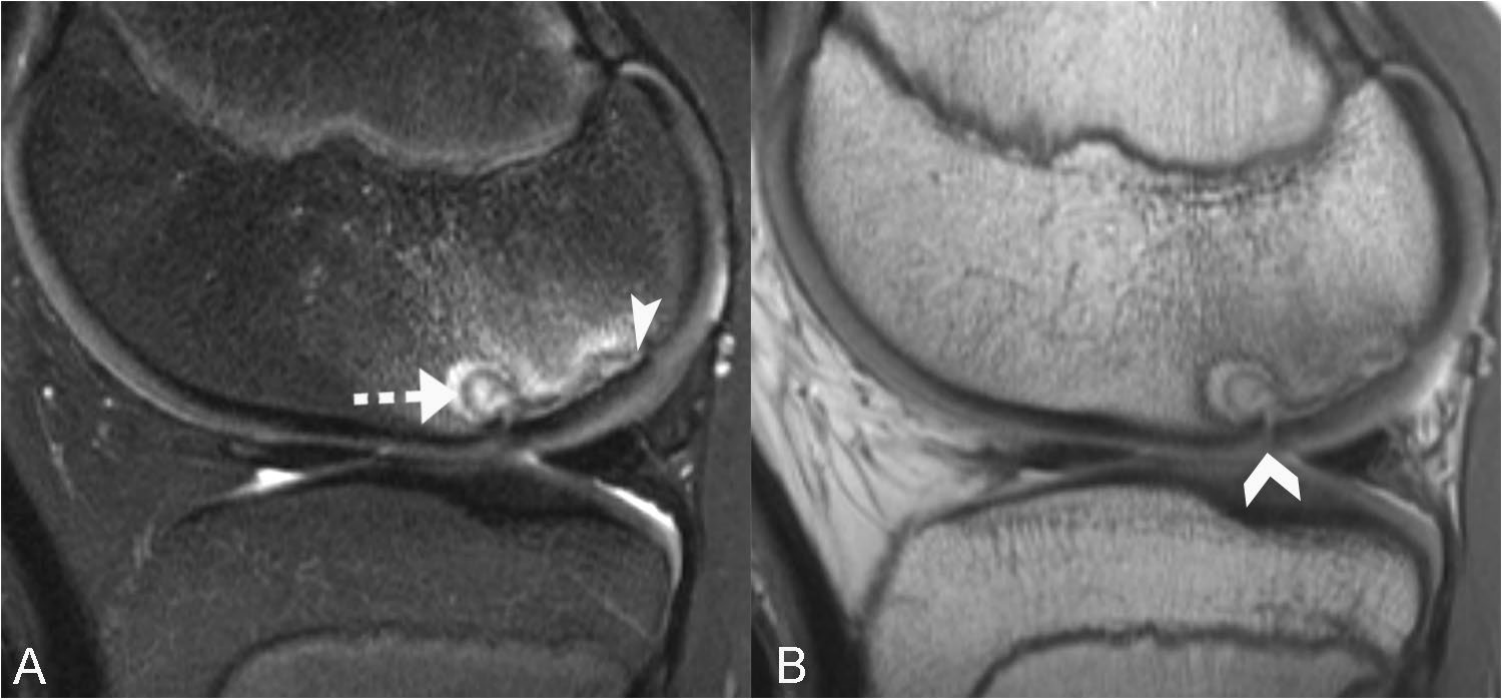
Unstable LFC OCD lesion in a 15-year-old skeletally immature male. Sagittal T2-weighted fat-suppressed (**A**) and intermediate-weighted (**B**) MR images show an intermediate signal intensity progeny-parent bone interface (arrowhead, **A**) that is defined by the underlying parent bone marginal rim. At the anterior margin of the OCD lesion, there is a 4.5-mm cyst (dashed arrow) that underlies a hyperintense cartilage fissure (chevron). A ballotable unstable OCD lesion was confirmed at arthroscopy.

**Fig. 4:**
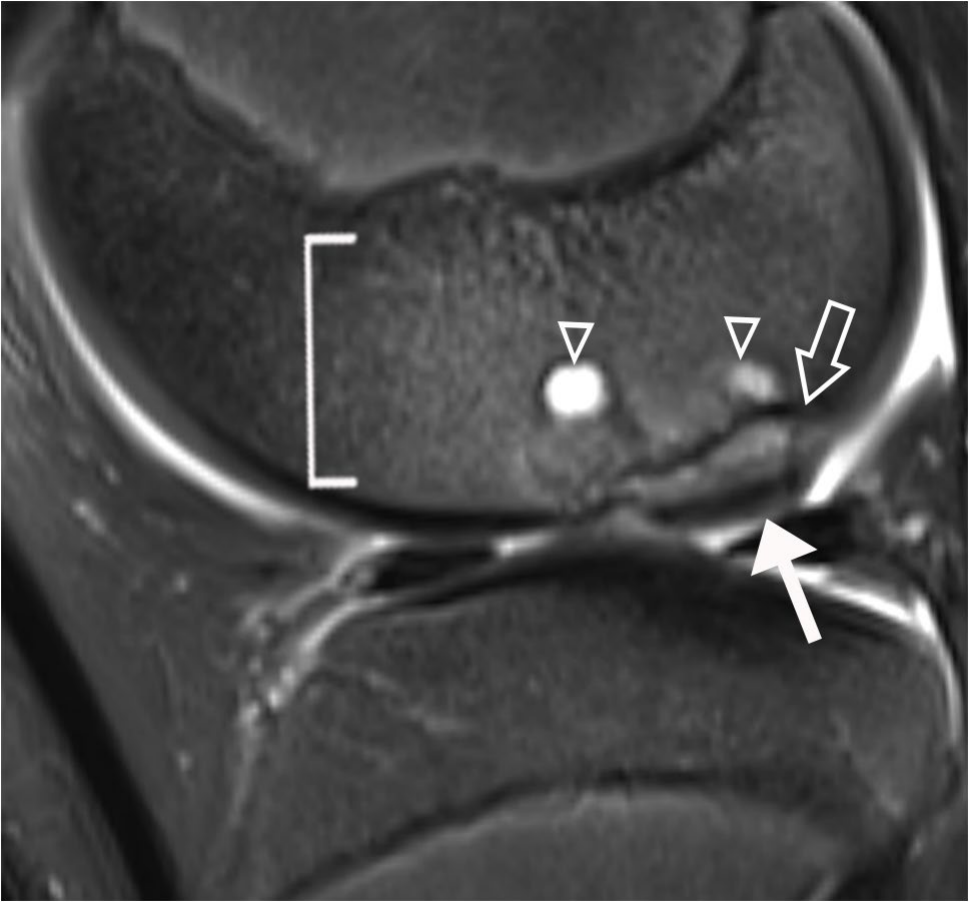
Unstable LFC OCD lesion in a 15-year-old skeletally immature male. Sagittal T2-weighted fat-suppressed MR image shows an abnormal convex surface contour (arrow), multiple cysts (triangles, largest measured 7 mm, not shown), parent bone marginal rim (block arrow), and extensive perilesional marrow edema (bracket). A protuberant unstable OCD lesion was confirmed at arthroscopy with overlying cartilage loss.

Indirect findings of lesion instability include the presence of a progeny-parent bone interface, cysts, altered articular surface curvature, parent bone marginal rim, and perilesional marrow edema. Interface was defined as a discrete separation between the progeny and the parent bone. If present, the extent (minimal, < 25%; moderate, 25–75%; or extensive, > 75%) and signal intensity (intermediate or fluid-high on T2-weighted images) of the interface were also recorded [21–23]. Cysts were defined as round foci with overall high signal intensity that are centered at the interface. If present, the total number and the diameter of the largest cyst were recorded [21–23] (Fig. 3). Articular surface curvature was categorized as maintained or altered, and the latter can convex or concave surface morphology [1]. Parent bone marginal rim was defined as a marginal band of low signal intensity on T2-weighted images [27]. If present, the extent (minimal, < 25%; moderate, 25–75%; or extensive, > 75%) of the rim was recorded. Perilesional marrow edema (low signal intensity on T1-weighted images and high signal intensity on fluid-sensitive images) was similarly subcategorized into mild (< 25%), moderate (25–75%), or extensive (> 75%) based on the extent of its craniocaudal involvement of the LFC (Fig. 4). For completeness, lesion volume is calculated using the ellipsoid formula (length × width × depth × 0.5) [28] and 3 dimensions of the lesion were measured by a research student (CY) with over 2 years of musculoskeletal research experience and under the supervision of the pediatric musculoskeletal radiologist (JCN).

### Clinical data and reference standards for OCD stability

The electronic medical records were reviewed to collect demographic and clinical information including age at the time of the MRI examination, laterality of the involved knee, duration of symptoms, BMI percentile (within 6 months of the MRI), lesion stability, and if available, operative findings. BMI was determined using the patient’s weight and height, which was then converted to percentile classification based on age- and sex-matched children from the CDC growth charts [29]. BMI percentile was classified as underweight if < 5%, normal weight if between 5 and < 85%, or as overweight or obese (hereafter, overweight/obese) if ≥ 85% [30]. For patients with bilateral lesions, the time interval between diagnoses was recorded. For lesions that underwent arthroscopy, the time interval between MRI and arthroscopy was recorded and for lesions that underwent conservative treatment, the time interval between initial MRI and latest follow-up was recorded.

**Fig. 5:**
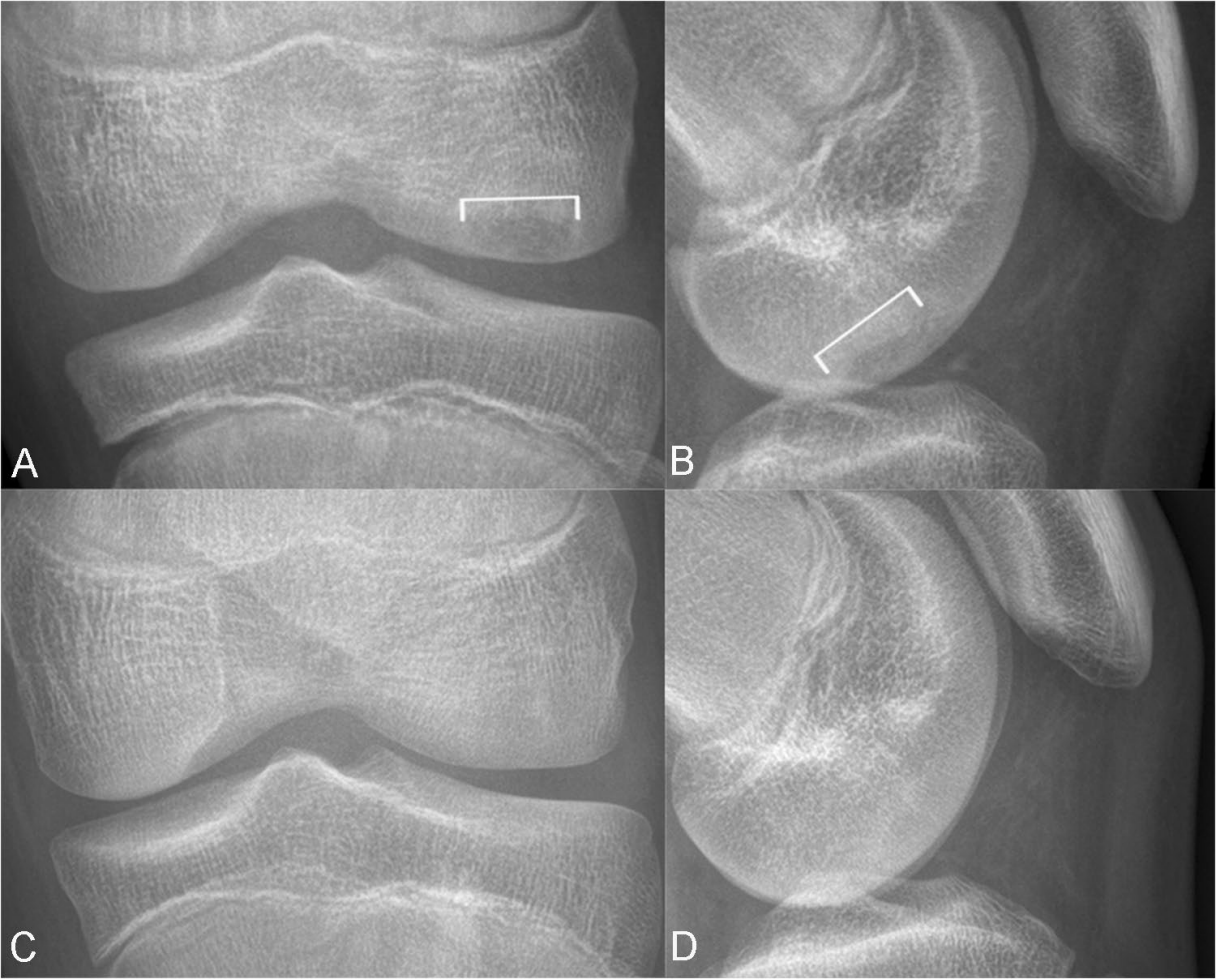
Conservatively treated LFC OCD lesion in a 13-year-old skeletally immature male. Anteroposterior (**A**, **C**) and lateral (**B**, **D**) radiographs at initial presentation (**A**, **B**) and 2 years later (**C**, **D**) show a less conspicuous progeny (brackets, A-B), no progeny-parent bone interface, or development of parent bone marginal sclerosis. Patient reported resolved symptoms.

Each LFC OCD lesion was classified as stable or unstable. For those patients who underwent arthroscopy, a LFC OCD lesion was considered unstable if the overlying cartilage was disrupted or if the lesion was ballotable upon probing [1, 22]. For those patients who underwent conservative management, a LFC OCD lesion was considered stable if symptoms improved or resolved, and follow-up imaging showed reduced lesion size, less conspicuous progeny, resolving and/or resolved parent bone marginal sclerosis on follow-up knee radiographs, reduced and/or resolved progeny-parent-bone interface, and/or cyst(s) on follow-up MRI examinations [20, 31–36] (Fig. 5).

### Statistical analysis

All statistical analyses were performed using R and R Studio (Version 1.1.423; R Foundation for Statistical Computing). A *P*-value < .05 was considered statistically significant. Interrater agreement for various MRI findings was evaluated using the Cohen’s kappa (*k*) with agreement categorized as slight (≤ .20), fair (.21–.40), moderate (.41–.60), substantial (.61–.80), almost perfect (≥ .81), and perfect (1.00) [37].

The Shapiro–Wilk test was used to assess the distribution of the variables. Continuous variables, if normally distributed, were reported as means and standard deviations (SD) and if not, reported as medians and interquartile ranges (IQR). Categorical variables were summarized using counts and percentages.

Comparisons between stable and unstable lesions were performed using the independent sample *t*, Mann–Whitney *U*, or the Fisher exact tests, depending on the distribution of data and sample size. Although univariable logistic regression analyses were performed to evaluate the utility of demographic and MRI findings for distinguishing lesion stability, the relatively small sample size compromised the reliability estimates for the multivariable logistic regression analysis increased the risk of model overfitting. Instead, the receiver operating characteristic (ROC) curve analysis provided a more robust and interpretable means of assessing the diagnostic performance of individual predictors while avoiding the assumptions and limitations of multivariable modeling in underpowered datasets. The area under the curve (AUC) was used as a summary measure of discriminative ability.

## Results

### Patients and lesions

The study group included 32 patients (21 male, 11 female). The mean age was 12.0 ± 2.0 years (range = 8.1–17.2). Five patients had bilateral lesions, which were diagnosed at a median duration of 207 days (IQR: 0–714 days) between knees. This yielded a total of 37 LFC OCD lesions in 37 knees, which included 26 stable (8 diagnosed at surgery and 18 underwent successful conservative treatment) and 11 unstable OCD lesions (8 diagnosed at surgery and 3 underwent conservative management). Three (27%) unstable lesions that did not undergo arthroscopy included bilateral knees in a patient where the family wanted to avoid surgery and 1 knee in a patient who reported persistent symptoms but declined follow-ups despite multiple reminders. Seven (18.9%) knees had discoid lateral menisci, and all had stable LFC OCD lesions.

Patients were older (mean age = 14.4 ± 1.9 vs. 11.9 ± 1.5 years, *P* < .01) and more likely to undergo arthroscopy (72.7% vs. 30.8%, *P* = .02) in the unstable than the stable groups. No significant differences were found between the groups in terms of sex (*P* = .57), laterality of the involved knee (*P* = .74), BMI percentile (*P* = .10), sports participation (*P* = .43), duration of symptoms prior to MRI (*P* = .59), days between MRI and arthroscopy (*P* = .21), or follow-up after MRI (*P* = .23) (Table 1).

**Table 1:** Characteristics of pediatric patients with lateral femoral condyle OCD lesions

Characteristic	All lesions (*n* = 37)	Stable lesions (*n* = 26)	Unstable lesions (*n* = 11)	*P*
Sex ^b^				.57
Male	21/32 (65.6)	19/26 (73.1)	7/11 (63.6)	
Female	11/32 (34.4)	7/26 (26.9)	4/11 (36.4)	
Age at time of MRI (y), mean ± SD (range) [count]	12.0 ± 2.0 (8.1–17.2) [37]	11.9 ± 1.5 (8.1–15.2) [26]	14.4 ± 1.9 (11.6–17.2) [11]	<.01^a^
Laterality				.74
Left	15/37 (40.5)	11/26 (42.3)	4/11 (36.4)	
Right	22/37 (59.4)	15/26 (57.7)	7/11 (63.6)	
BMI percentile classification				.10
Underweight (< 5%)	1/37 (2.7)	0/26 (0)	1/11 (9.1)	
Normal weight (5 to < 85%)	24/37 (64.9)	18/26 (69.2)	6/11 (54.5)	
Overweight/obese (≥ 85%)	12/37 (32.4)	8/26 (30.8)	4/11 (36.4)	
Sports participation^c^	33/37 (89.2)	24/26 (92.3)	9/11 (81.8)	.43
One	11/33 (33.3)	7/24 (29.2)	4/9 (44.4)	
Multiple	22/33 (66.7)	17/24 (70.8)	5/9 (55.6)	
Duration (d), median (IQR) [count]				
Symptoms prior to MRI	81 (30–295) [35]	81 (26–146) [25]	78 (35–336) [10]	.59
Between MRI and arthroscopy	44 (27.5–59.5) [16]	35 (22.5–51) [8]	53.5 (36.5–64.5) [8]	.21
Follow-up after MRI	268 (164–660) [37]	252 (170–473) [26]	660 (159–1209) [11]	.23
Arthroscopy	16/37 (43.2)	8/26 (30.8)	8/11 (72.7)	.02^a^

*d*, day(s); *IQR*, interquartile range; *OCD*, osteochondritis dissecans; *SD*, standard deviation; *y*, year(s)

If not otherwise specified, values are numerator and denominator with percentage in parentheses

^a^Values are statistically significant

^b^Reported as patients (*n* = 32) under “all lesions” and as OCD lesions under “stable lesions (*n* = 26)” and “unstable lesions (*n* = 11)”

^c^Twenty-eight children (33 lesions) reported participation in 14 different sports with 18 (22 lesions) in multiple sports (10 in 2 sports, 7 in 3 sports, and 1 in 4 sports), yielding a total of 53 sports. In descending order, sports included basketball (14/28 children, 50.0%), baseball (9/28, 32.1%), soccer (6/28, 21.4%), swimming (5/28, 17.9%), football (4/28, 14.3%), softball (3/28, 10.7%), martial arts, volleyball, running, and wrestling (2 each/28, 7.1%), boxing, gymnastics, cheerleading, and lacrosse (1 each/28, 3.6%)

### MRI findings

Table 2 summarizes the MRI findings based on consensus readings with corresponding interreader agreement. Interreader agreement was substantial to perfect (*k* range: .61–1.00) with the exception of moderate agreement (*k* range: .53–.58) for ossification front, interface signal intensity, extent of parent bone marginal rim, and perilesional marrow edema. There was a significant (*P* = .02) association between skeletal maturity and lesion stability with over a third (36.4%m 4/1) of unstable lesions found in skeletally maturing and mature knees whereas most (96.2%, 25/26) of stable lesions were found in skeletally immature knees. Larger lesions were found in the unstable than the stable groups (436.4 mm^3^, IQR 371–515 vs. 230.8 mm^3^, IQR 156–362; *P* < .01).

**Table 2:** MRI findings from children with lateral femoral condyle OCD lesions with interreader agreement

Characteristic or finding	All lesions (*n* = 37)	Stable lesions (*n* = 26)	Unstable lesions (*n* = 11)	*P*	*k*
Joint effusion	2/37 (5.4)	0/26 (0)	2/11 (18.2)	.08	.65
Skeletal maturity					
Immature Maturing + mature	32/37 (86.5) 5/37 (13.5)	25/26 (96.2) 1/26 (3.8)	7/11 (63.6) 4/11 (36.4)	.02^a^	.81
OCD lesion volume (mm^3^), median (IQR)	288.1 (203–376)	230.8 (156–362)	436.4 (371–515)	<.01^a^	NA
Osteochondral defect	0	0	0	>.99	1.00
Intraarticular body	2/37 (5.4)	0/26 (0)	2/11 (18.2)	.08	.65
Cartilage alteration^b^	12/37 (32.4)	2/26 (7.7)	10/11 (90.9)	<.01^a^	.69
Cartilage alteration signal intensity^b^					
Hyperintense Hypointense Both hyper- and hypointense	2/12 (16.7) 2/12 (16.7) 8/12 (66.7)	1/2 (50.0) 1/2 (50.0) 0/2 (0.0)	1/10 (10.0) 1/10 (10.0) 8/10 (80.0)	.09	.81
Cartilage alteration location(s)^b^					
Single Multiple	6/12 (66.7) 6/12 (33.3)	2/2 (100.0) 0/2 (0)	4/10 (60.0) 6/10 (40.0)	.12	.69
Bone plate disruption^c^					
Marginal–focal Central–multifocal	2/5 (40.0) 3/5 (60.0)	1/1 (100.0) 0/1 (0)	1/4 (25.0) 3/4 (75.0)	.40	.61
Ossification front^d^					
Normally located Receded	6/32 (18.7) 26/32 (81.3)	3/25 (22.0) 22/25 (88.0)	3/7 (42.9) 4/7 (57.1)	.10	.54
Interface, progeny-parent bone	15/37 (40.5)	6/26 (23.1)	9/11 (81.2)	<.01^a^	.67
Interface extent^e^					
Minimal (< 25%) Moderate (25–75%) Extensive (> 75%)	1/15 (6.7) 3/15 (20.0) 11/15 (73.3)	1/6 (16.2) 1/6 (16.2) 4/6 (66.7)	0/9 (0) 2/9 (22.2) 7/9 (77.8)	.72	.62
Interface signal intensity^e^					
Intermediate Fluid-high	8/15 (55.3) 7/15 (60.8)	4/6 (33.3) 2/6 (66.7)	4/9 (44.4) 5/9 (55.6)	.61	.44
Cyst(s)	26/37 (70.3)	16/26 (61.5)	10/11 (90.9)	.11	NA^b^
Number of cysts^f^					
≤ 5 > 5	24/26 (92.3) 2/26 (7.7)	15/16 (93.8) 1/16 (6.2)	9/10 (90.0) 1/10 (10.0)	.72	NA^b^
Largest cyst diameter^f^					
≤ 5 mm > 5 mm	25/26 (96.2) 1/26 (3.8)	16/16 (100.0) 0/16 (0)	9/10 (90.0) 1/10 (10.0)	.19	NA^b^
Altered articular surface curvature	14/37 (37.8)	6/26 (23.1)	8/11 (72.7)	<.01^a^	.70
Parent bone marginal rim	12/37 (32.4)	3/26 (11.5)	9/11 (81.8)	<.01^a^	.80
Marginal rim, extent^g^					
Minimal (< 25%) Moderate (25–75%) Extensive (> 75%)	1/12 (8.3) 4/12 (33.3) 7/12 (58.3)	0/3 (0) 1/3 (33.3) 2/3 (66.7)	1/9 (11.1) 3/9 (33.3) 5/9 (55.6)	1.00	.53
Perilesional marrow edema, extent^h^					
Mild (< 25%) Moderate (25–75%) Extensive (> 75%)	16/37 (43.2) 10/37 (27.0) 11/37 (29.7)	16/26 (61.5) 7/26 (27.0) 3/26 (11.5)	0/11 (0) 3/11 (27.3) 8/11 (72.7)	<.01^a^	.58

*OCD, osteochondritis dissecans; IQR*, interquartile range; *NA*, not applicable

Unless otherwise specified, values are numerator and denominator with percentage in parentheses

^a^Values are statistically significant

^b^Among the 12 LFC OCD lesions with cartilage alterations, 6 (50.0%) had single and 6 (50.0%) had multiple sites (2 in 2 lesions; 3 in 3 lesions; and 4 in 1 lesion), which were most commonly located along the anterior and the medial margins (each 30.4%, 7/23) of the lesion, followed by lateral (21.7%, 5/23) and posterior (17.4%, 4/23) margins.

^c^Only assessed for lesions in maturing or mature knees

^d^Only assessed for lesions in skeletally immature knees

^e^Only assessed for lesions with an interface

^f^Only assessed for lesions with cyst(s)

^g^Only assessed for lesions with parent bone marginal rim

^h^Additional comparison between extensive (29.7%, 11/37) and mild or moderate (70.3, 26/37) perilesional marrow edema remained significant (*P* <.01)

No LFC OCD lesion was completely detached to leave behind an osteochondral defect. Both knees (5.4%, 2/37) with intraarticular bodies had unstable OCD lesions (Fig. 2). Cartilage alteration was more common among lesions in the unstable than the stable groups (90.9% vs. 7.7%, *P* < .01). Among the indirect findings, the presence of a progeny-parent bone interface (81.2% vs. 23.1%, *P* < .01), altered articular surface curvature (72.7% vs. 23.1%, *P* < .01), and parent bone marginal rim (81.8% vs. 11.5%, *P* < .01) were more common among lesions in the unstable than the stable groups. The distribution of perilesional marrow edema differed between groups with most lesions in the unstable group (72.7%, 8/11) had extensive edema whereas most lesions in the stable group (61.5%, 16/26) had mild edema (*P* < .01). No significant differences were found between the groups in terms the presence of joint effusion (*P* = .08), intraarticular body (*P* = .08), bone plate disruption (*P* = .40), location of the ossification front (*P* = .10), or cysts (*P* = .11).

### Identification of predictors of lesion instability

Univariable logistic regression analyses found cartilage alteration had the highest odds ratio for lesion instability (OR, 120.0, 95% CI = 14.1–2970.1, *P* < .01), followed by parent bone marginal rim (OR, 34.5, 95% CI = 5.9–322.7, *P* < .01), extensive perilesional marrow edema (OR, 20.4, 95% CI = 3.9–149.0, *P* < .01), skeletal maturity (OR, 14.3, 95% CI = 1.37–149.2, *P* = .03), progeny-parent bone interface (OR, 11.2, 2.4–68.1, *P* < .01), altered articular surface curvature (OR, 8.9, 95% CI = 1.9–51.9, *P* < .01), and chronologic age (OR, 2.3, 95% CI = 1.4–4.3, *P* < .01) (Table 3). ROC curve analyses found that cartilage alteration had the highest discriminative performance (AUC = .92, 95% CI = .81–1.00) with sensitivity of 91% and specificity of 92% for predicting unstable LFC OCD lesions. No single finding was 100% sensitive, but skeletal maturity was 100% specific, but only 18% sensitive, for predicting lesion instability (AUC = .59, 95% CI = .47–.71) (Table 4).

**Table 3:** Univariable regression analyses for predicting instability of lateral femoral condyle OCD lesions.

Characteristic or finding	Level	Reference	Univariable	
			OR (95% CI)	*P*
Chronologic age	Per 1-year increase	NA	2.3 (1.4–4.3)	<.01^a^
Sex	Female	Male	1.6 (0.3–7.0)	.57
Skeletal maturity	Maturing and mature	Immature	14.3 (1.37–149.2)	.03^a^
OCD lesion volume	Per 1-mm^3^ increase	NA	1.0 (1.0–1.1)	.64
Cartilage alteration	Present	Absent	120.0 (14.1–2970.1)	<.01^a^
Interface, progeny-parent bone^b^	Present	Absent	11.2 (2.4–68.1)	<.01^a^
Altered articular surface curvature	Altered	Maintained	8.9 (1.9–51.9)	<.01
Parent bone marginal rim^b^	Present	Absent	34.5 (5.9–322.7)	<.01^a^
Extensive perilesional marrow edema	Extensive	Mild or moderate	20.4 (3.9–149.0)	<.01^a^

^a^Values are statistically significant

^b^Extent of the interface and parent bone marginal rim were not evaluated in the regression models given their associations with presence of interface and rim, respectively

*CI, confidence interval; NA*, not applicable; *OCD*, osteochondritis dissecans; *OR*, odds ratio

**Table 4:** ROC curve performances for predicting instability of lateral femoral condyle OCD lesions.

Characteristic or finding	AUC (95% CI)	Sensitivity	Specificity
Older chronologic age	0.83 (0.67–0.99)	0.64	0.96
Skeletal maturity	0.59 (0.47–0.71)	0.18	1.00
Cartilage alteration	0.92 (0.81–1.00)	0.91	0.92
Interface, progeny-parent bone	0.77 (0.61–0.93)	0.73	0.81
Altered surface curvature	0.75 (0.59–0.91)	0.73	0.77
Parent bone marginal rim	0.85 (0.72–0.99)	0.82	0.89
Perilesional marrow edema, extensive (> 75%)	0.81 (0.65–0.96)	0.73	0.89

*AUC*, area under the curve; *CI*, confidence interval; *OCD*, osteochondritis dissecans; *ROC*, receiver operating characteristic

## Discussion

Our results found several findings that were more common with unstable than stable LFC OCD lesions. In contrast to MFC lesions, the presence of cysts was not predictive of lesion instability. As with lesions at other anatomic locations, unstable lesions were more likely to be found among older children and in skeletally mature knees. Several MRI findings differed between stable and unstable lesions, but the presence of cartilage alteration had the highest discriminative performance.

Lesions over the LFC are far less common when compared to those over the MFC [5]. This has led to the use of MRI criteria originally derived from studies that used all or predominantly MFC lesions [8, 22] to characterize LFC lesions, but without additional location-specific information on diagnostic performance. Results from our study further this line of inquiry and demonstrate that most direct and indirect MRI findings for instability overlap between MFC and LFC lesions, but important differences do exist. Specifically, the presence of cysts was found in over two-thirds of all LFC OCD lesions in our study group, but did not distinguish between stable and unstable lesions. Additionally, although intraarticular bodies have been reported to associate with unstable OCD lesions of the capitellum [12, 38–41], these bodies are uncommon in the knee joint. In a recent study on MFC OCD lesions, none of the 69 MRI examinations had intraarticular bodies [8]. In our group of LFC OCD lesions, two knees had intraarticular bodies and both had unstable lesions, which accounted for over 18% of all unstable lesions. Other indirect MRI findings of lesion instability overlap with those previously reported for MFC OCD lesions, which included the presence of progeny-parent bone interface, altered articular surface curvature, parent bone marginal rim, and extensive perilesional bone marrow edema.

Older chronologic age of the patient and regional skeletal maturity of the knee were predictors of lesion instability, which support our current understanding on the maturation-dependent differences in healing and remodeling potential, which is independent of location [3, 6, 10, 11, 18, 27, 31–33, 42–44]. This is because in the skeletally immature, the vascularized unossified chondroepiphysis and the secondary physis can facilitate regional healing and remodeling, which is diminished with progressive skeletal maturation [26, 45, 46]. In our study group, the mean chronologic age was over 2 years higher for lesions in the unstable than the stable groups and both skeletally mature knees had unstable LFC OCD lesions (Fig. 2).

In our study group, cartilage alteration had the highest discriminative performance for predicting lesion instability with over 90% sensitivity and specificity. This is concordant with previously published data on MFC OCD lesions [8]. It is well recognized that sites of cartilage alteration are often identified over the peripheral circumference of the OCD lesion [20] and in our group of LFC OCD lesions, the most common sites of cartilage breach occurred along the anterior and medial margins. Although the presence of both hyper- and hypointense signal alterations and multiple sites of involvement were more common with unstable than stable lesions, these findings did not reach statistical significance, possibly due to the size of our study group. Classically, a cartilage fissure is defined as a band of hyperintense signal, but more recently, hypointense signal, either in isolation (also known as a dark cartilage lesion or “omen sign”) or bordering the hyperintense signal, is increasingly recognized [8, 21–23, 25, 47]. Currently, it is uncertain whether this hypointense signal reflects early cartilage degeneration [47] or fissures without intervening joint fluid. Future prospective studies with a larger number of patients and with arthroscopic correlation are necessary to further characterize and validate these observations on preoperative MRI.

Our study has several limitations. First, the retrospective study design hinders the gathering of additional clinical, imaging, and operative data. The use of arthroscopy as a reference standard can be problematic if intraoperative assessment is partially influenced by the initial MRI report and/or clinical assessment. Conversely, the use of patient-reported resolution of symptoms can be subjective and variable among patients. However, all patients in our study group also had additional follow-up imaging that showed findings of lesion healing (Fig. 5), and all included patients had a minimal follow-up duration of 90 days or more to establish interval change. Second, the inclusion of both 1.5-T and 3-T MRI examinations introduced some heterogeneity to our study group, but this approach represents the routine clinical practice and makes our results more generalizable. Although our sample size is small, it remains the only study that investigates various MRI findings exclusively using LFC OCD lesions. Third, while we excluded patients with preoperative MRI examinations that were acquired more than 90 days before arthroscopy, we cannot exclude the possibility that some lesions may progress or heal, potentially leading to false-negative and false-positive diagnoses that can affect the calculated diagnostic performance of preoperative MRI. Finally, the wide confidence intervals reflect the small number of unstable lesions and sparse data in some of the imaging finding categories, which can yield unstable effect estimates and potentially inflated effect sizes. Thus, future studies with a larger number of unstable lesions are necessary to further validate these findings.

## Conclusion

Although various MRI findings can associate with unstable LFC OCD lesions, most are concordant with those used with MFC OCD lesions. One exception is the presence of cysts, which did not significantly differ between stable and unstable lesion groups. Cartilage alteration had the highest discriminative performance and is more commonly found over the anterior and medial margins of these lesions.

## Data Availability

Data is available upon reasonable request.

## Abbreviations

AUC: Area under the curve
CI: Confidence interval
HIPPA: Health Insurance Portability and Accountability
IQR: Interquartile range
IRB: Institutional Review Board
LFC: Lateral femoral condyle
MFC: Medial femoral condyle
MRI: Magnetic resonance imaging
ms: Millisecond
NA: Not applicable
OCD: Osteochondritis dissecans
OR: Odds ratio
ROC: Receiver operating characteristic
SD: Standard deviation
TE: Echo time
TR: Repetition time
